# Supplementary material for: SHMT2 reduces fatty liver but is necessary for liver inflammation and fibrosis in mice
Source: Commun Biol. 2024 Feb 12;7:173. doi: 10.1038/s42003-024-05861-y (PMC10861579; doi:10.1038/s42003-024-05861-y)
Supplement: Supplementary file 2 — Supplementary Information [file 42003_2024_5861_MOESM2_ESM.pdf]

SHMT2 reduces fatty liver but is necessary for liver inflammation and fibrosis in mice

Guohua Chen[1], Guoli Zhou[2], Lidong Zhai[3], Xun Bao[4], Nivedita Tiwari[5], Jing Li[4], Emilio Mottillo[5,6] and Jian Wang[1*]

[1] Department of Pathology, Wayne State University School of Medicine, Detroit, MI 48202, USA

[2] Biomedical Research Informatics Core, Clinical and Translational Sciences Institute, Michigan State University, East Lansing, MI 48824 USA

[3] Department of Pathology, University of Michigan, Ann Arbor, MI 48109, USA

[4] Department of Oncology, Wayne State University School of Medicine, Detroit, MI 48202, USA

[5] Hypertension and Vascular Research Division, Henry Ford Hospital, Detroit. MI 48202 USA

[6] Department of Physiology, Wayne State University School of Medicine, Detroit, MI 48202, USA

Supplementary Figure 1

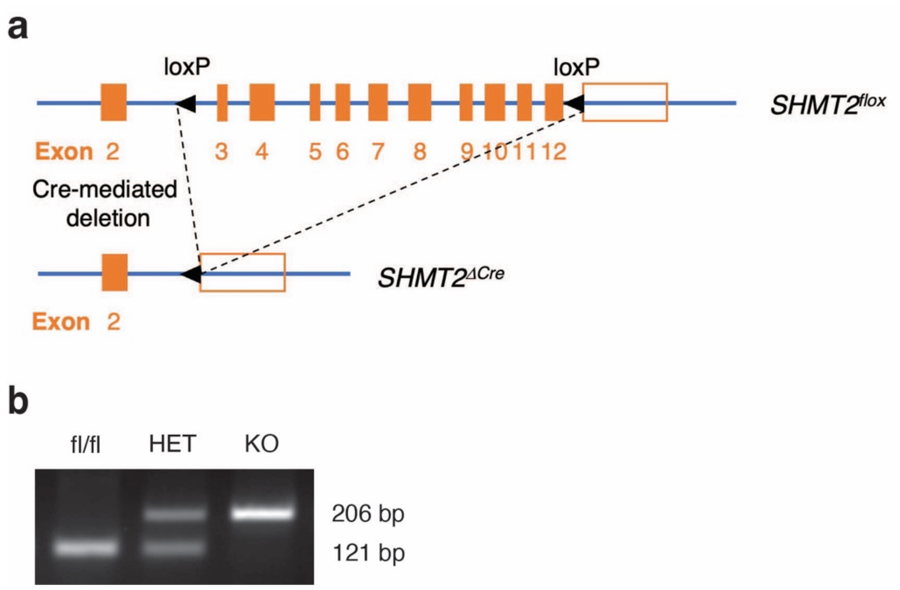

Fig. S1. Targeted deletion of SHMT2 gene in mice using Cre-loxP recombination.
a) Schematic diagram of the floxed mouse SHMT2 gene loci before (*SHMT2*^{flox}, top) and after (*SHMT2*$^{\Delta Cre}$, bottom) Cre-mediated deletion. **b)** PCR analysis of the genomic DNA from the mice with floxed SHMT2 gene (fl/fl) or heterozygous (HET) or homozygous (KO) deletion of the SHMT2 gene. The sizes of the amplicons are indicated on the right.

Supplementary Figure 2

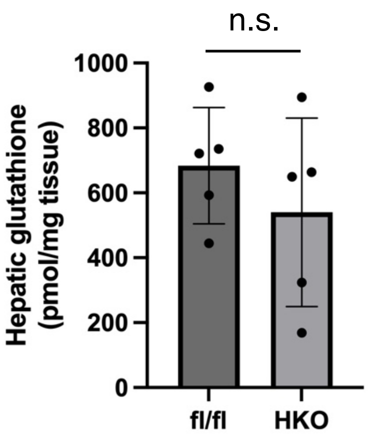

Fig. S2. The deletion of SHMT2 has no significant effect on liver glutathione levels.
The liver metabolites were extracted from SHMT2 wild-type (fl/fl) and knockout (HKO) mice and analyzed for total glutathione levels using LC-MS/MS. (Mean±SD; n.s., not significant; t-test)

Supplementary Figure 3

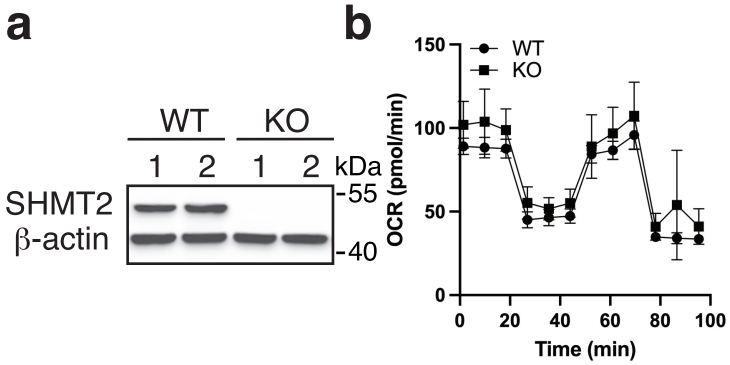

Fig. S3. The effect of SHMT2 knockout on the oxygen consumption rate of AML12 cells. **a)** Western blot analysis of the indicated proteins in the SHMT2 wide-type (WT) and knockout (KO) AML12 cells. **b)** Analysis of the oxygen consumption rate (OCR) of the indicated AML12 cells using a Seahorse analyzer.

Supplementary Figure 4

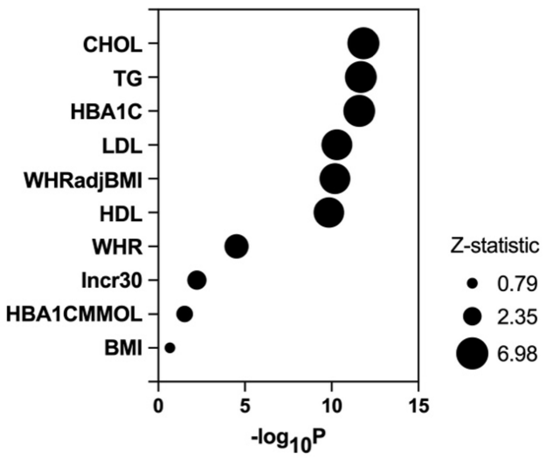

Fig. S4. The association of single nucleotide polymorphism in the SHMT1 gene with human metabolic syndrome.

Supplementary Figure 5

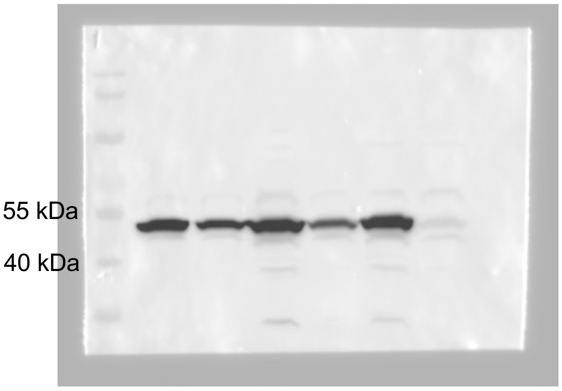

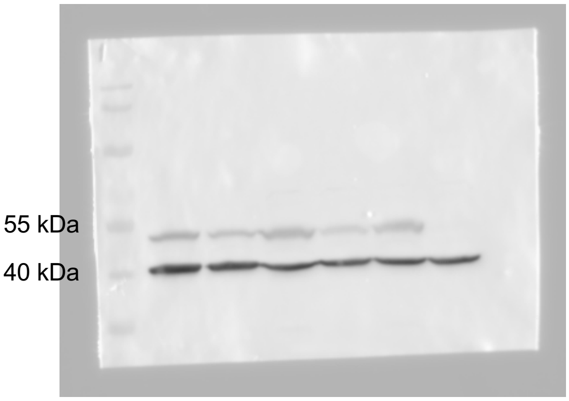

a) Uncropped gel images of Figure 1a

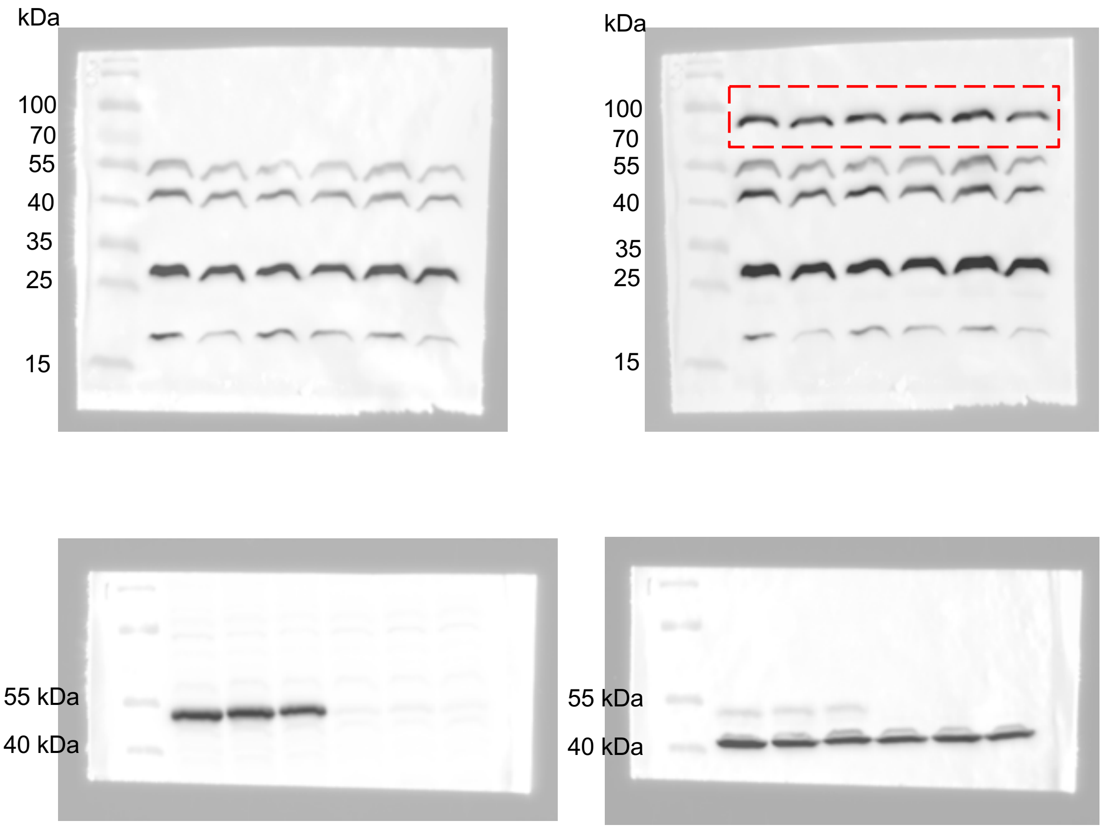

b) Uncropped gel images of Figure 5a

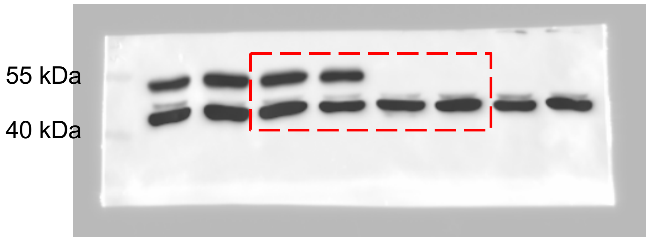

c) Uncropped gel image of Figure 5b

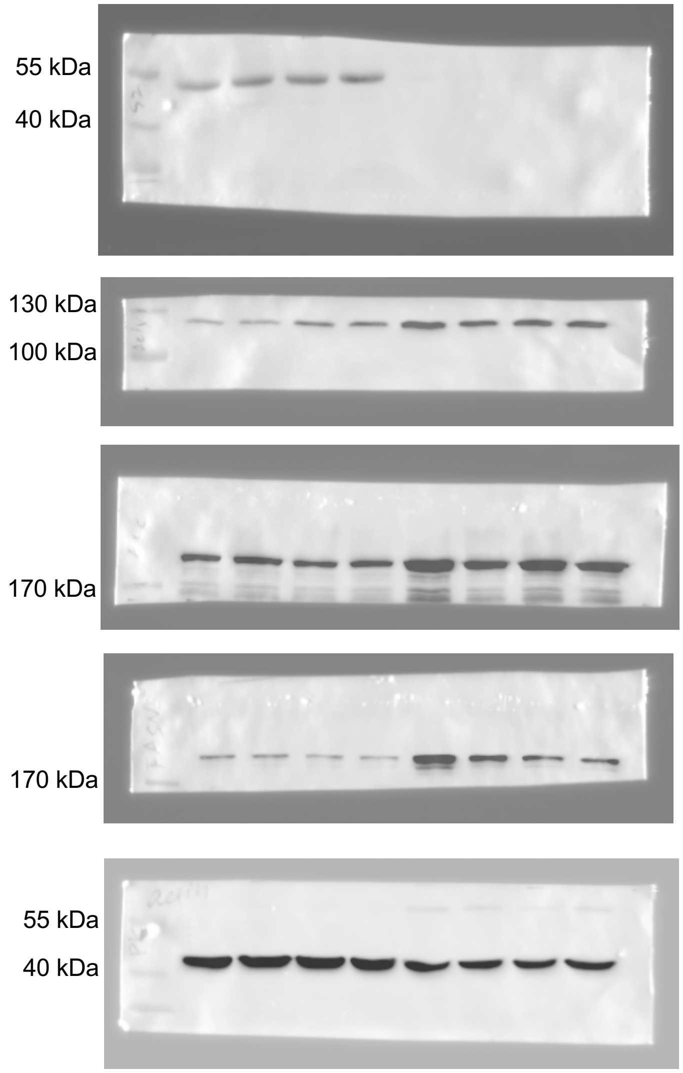

d) Uncropped gel images of Figure 6e

55 kDa
40 kDa

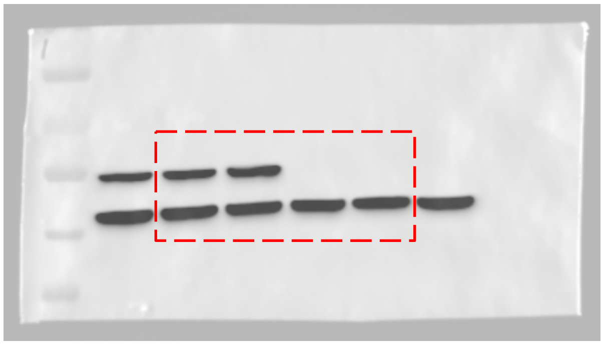

d) Uncropped gel images of Supplementary Figure 3a

Table S1: SHMT2 SNP is linked to metabolic traits in humans.

Phenotype	SNP Variant#	Sample size	Z-statistic	P-value
TG	7	1,162,680	5.40	3.36E-08
HDL	7	1,123,112	4.83	6.93E-07
WHR	7	539,439	3.28	5.22E-04
HBA1C	7	419,439	3.07	1.05E-03
LDL	7	1,144,184	2.89	1.90E-03
CHOL	7	1,194,060	2.88	1.96E-03
WHRadjBMI	7	534,261	2.02	2.16E-02
BMI	7	547,953	2.00	2.29E-02
HBA1CMMOL	7	7,325	1.84	3.30E-02
Incr30	1	4,447	1.79	3.66E-02

Table S2: SHMT1 SNP is linked to metabolic traits in humans.

Phenotype	SNP Variant#	Sample size	Z-statistic	P-value
CHOL	34	1,393,255	6.98	1.49E-12
TG	34	1,487,737	6.93	2.10E-12
HBA1C	33	587,581	6.90	2.59E-12
LDL	34	1,673,533	6.47	5.06E-11
WHRadjBMI	31	808,835	6.43	6.56E-11
HDL	34	1,329,001	6.30	1.46E-10
WHR	31	880,504	4.01	3.09E-05
Incr30	3	4,447	2.51	5.97E-03
HBA1CMMOL	30	8,088	1.88	3.02E-02
BMI	32	1,157,212	0.79	2.14E-01

Table S3: qPCR primers for mouse genes

Gene	Forward sequence	Reverse Sequence
Acly	AGGAAGTGCCACCTCCAACAGT	CGCTCATCACAGATGCTGGTCA
Acaca	GTTCTGTTGGACAACGCCTTCAC	GGAGTCACAGAAGCAGCCCATT
Acacb	AGAAGCGAGCACTGCAAGGTTG	GGAAGATGGACTCCACCTGGTT
Fasn	CACAGTGCTCAAAGGACATGCC	CACCAGGTGTAGTGCCTTCCTC
Scd1	GCAAGCTCTACACCTGCCTCTT	CGTGCCTTGTAAGTTCTGTGGC
Ccl2	GCTACAAGAGGATCACCAGCAG	TCTGGACCCATTCCTTCTTGG
Adgre1	CGTGTTGTTGGTGGCACTGTGA	CCACATCAGTGTTCCAGGAGAC
TNF	GGTGCCTATGTCTCAGCCTCTT	GCCATAGAACTGATGAGAGGGAG
Col1a1	CCTCAGGGTATTGCTGGACAAC	CAGAAGGACCTTGTTTGCCAGG
Col3a1	GACCAAAAGGTGATGCTGGACAG	CAAGACCTCGTGCTCCAGTTAG
Des	GCGGCTAAGAACATCTCTGAGG	ATCTCGCAGGTGTAGGACTGGA
Timp1	TCTTGGTTCCCTGGCGTACTCT	GTGAGTGTCACTCTCCAGTTTGC
PPIA	GTGGTCTTTGGGAAGGTGAA	TTACAGGACATTGCGAGCAG
