# Supplementary material for: SHMT2 reduces fatty liver but is necessary for liver inflammation and fibrosis in mice
Source: Commun Biol. 2024 Feb 12;7:173. doi: 10.1038/s42003-024-05861-y (PMC10861579; doi:10.1038/s42003-024-05861-y)
Supplement: Supplementary file 3 — Description of Supplementary Materials [file 42003_2024_5861_MOESM3_ESM.docx]

**Description of Additional Supplementary Files**

**File name:** Supplementary Data 1

**Description:** Normalized metabolite levels in the mouse livers

**File name:** Supplementary Data 2

**Description:** Fold change of liver metabolites (HKO vs. fl/fl)

**File name:** Supplementary Data 3

**Description:** Fold change in gene expression levels in HKO mice livers on AMLN diet compared to control mice

**File name:** Supplementary Data 4

**Description**: DEG identified in HKO mouse livers on AMLN diet compared to the control

**File name:** Supplementary Data 5

**Description:** Numerical source data
